# Mulberry Anthocyanin Extract Ameliorates Oxidative Damage in HepG2 Cells and Prolongs the Lifespan of *Caenorhabditis elegans* through MAPK and Nrf2 Pathways

**DOI:** 10.1155/2017/7956158

**Published:** 2017-06-21

**Authors:** Fujie Yan, Yushu Chen, Ramila Azat, Xiaodong Zheng

**Affiliations:** ^1^Department of Food Science and Nutrition, Zhejiang University, Hangzhou 310058, China; ^2^Zhejiang Key Laboratory for Agro-food Processing, Zhejiang University, Hangzhou 310058, China; ^3^Fuli Institute of Food Science, Zhejiang University, Hangzhou 310058, China

## Abstract

Mulberry anthocyanins possess many pharmacological effects including liver protection, anti-inflammation, and anticancer. The aim of this study was to evaluate whether mulberry anthocyanin extract (MAE) exerts beneficial effects against oxidative stress damage in HepG2 cells and *Caenorhabditis elegans.* In vitro, MAE prevented cytotoxicity, increased glucose consumption and uptake, and eliminated excessive intracellular free radicals in H_2_O_2_-induced cells. Moreover, MAE pretreatment maintained Nrf2, HO-1, and p38 MAPK stimulation and abolished upregulation of p-JNK, FOXO1, and PGC-1*α* that were involved in oxidative stress and insulin signalling modulation. In vivo, extended lifespan was observed in *C. elegans* damaged by paraquat in the presence of MAE, while these beneficial effects were disappeared in *pmk-1* and *daf-16* mutants. PMK-1 and SKN-1 were activated after exposure to paraquat and MAE suppressed PMK-1 activation but enhanced SKN-1 stimulation. Our findings suggested that MAE recovered redox status in HepG2 cells and *C. elegans* that suffered from oxidative stress, which might be by targeting MAPKs and Nrf2.

## 1. Introduction

Generation of excessive reactive oxygen species (ROS) results in oxidative stress that disturbs intracellular redox status homeostasis and induces cell apoptosis or death [1]. Prolonged oxidative stress impairs normal functions of cells, tissues, and organs, potentially accelerating the process of some diseases such as cancer, hepatopathy, diabetes, and obesity [2]. Therefore, it seems that oxidative stress becomes a critical target in the prevention of chronic diseases.

Nuclear factor erythroid 2-related factor 2 (Nrf2), a short-lived protein, allows adaption and survival under stress by regulating expression of genes and proteins with diverse cytoprotective functions including anti-inflammatory, antioxidant, and detoxification enzymes. Kelch-like ECH-associated protein 1 (Keap1) is a negative regulator of Nrf2, contributing to its degradation under normal conditions. Keap1-Nrf2 interactions are disrupted by shear stress or other physiological stimuli, causing nuclear accumulation of Nrf2, leading to the transcription of its target genes such as heme oxygenase-1 (HO-1), g-glutamate-cysteine ligase (GCL), and NAD(P)H:quinone oxidoreductase 1 (NQO1) [3, 4]. HO-1 acts as a rate-limiting enzyme in heme metabolism, and GCL catalyzes the first and rate-limiting step of GSH biosynthesis [5, 6]. NQO1 protects cells from oxidative damage through catalyzing quinone detoxification [7]. Therefore, Nrf2 plays a crucial role in cellular redox homeostasis, mitochondrial health, and fatty acid oxidation. Moreover, MAPKs can affect Nrf2 activity and are involved in oxidative stress regulation [8–10].

Anthocyanins belong to the family of flavonoids but are different from other flavonoids because of their flavylium cation structures. They occur in berries, deep-color fruit, and grains that are well represented in the human diet. Mulberry fruit, which is abundant in water soluble anthocyanins such as cyanidin-3-glucoside and cyanidin-3-rutinoside, has been traditionally used in Chinese medicines due to its pharmacological effects [11]. Mulberry possesses many beneficial actions including antioxidation, anti-inflammation, liver protection, and anticancer either in cultured cells or in animal models, which is partly associated with its capacity of free radical scavenging [12]. Attributed to the special molecular structure, anthocyanins show great antioxidant properties in in vitro analyses such as ferric reducing antioxidant potential (FRAP) and oxygen radical absorbance (ORAC) [13]. Although studies reported that the bioavailability of anthocyanins was low, which meant tiny related substance could be detected in plasma and urine [14], anthocyanin administration indeed obviously increased plasma antioxidant ability in Wistar rats, suggesting that some antioxidant compounds in blood are activated after anthocyanin digestion and absorption [15]. Protective roles of mulberry anthocyanins in the liver have been reported in both cells and animals, which are partly connected with oxidative alleviation [16–18]. Nevertheless, researches about protective effectiveness and possible mechanisms at molecular levels of MAE on cells and *C. elegans* under conditions of oxidative stress are insufficient.

In this study, in vitro, we aimed to investigate MAE-based protection against H_2_O_2_-induced oxidative stress in HepG2 cells and confirm whether this effect depends upon MAPK and Nrf2 modulation. Furthermore, we estimated the impact of MAE on paraquat-mediated oxidative injury in *Caenorhabditis elegans* in vivo. Results from this model organism may be more relevant for an evaluation of the biological actions of anthocyanins in humans.

## 2. Material and Methods

### 2.1. Preparation of MAE

Mulberry anthocyanins were isolated and purified as previously described [19]. Briefly, mulberry fruit (*Morus alba* L.) was purchased from the local market (Hangzhou, China) and extracted with a 3-fold volume of 95% ethanol containing 1% HCl for 24 h at 4°C. Filtered fluid was evaporated at 38°C, and then, the concentrates were loaded onto an equilibrated AB-8 macroporous resin column orderly eluted with 1% formic acid in 80% methanol for further purification. MAE was obtained by lyophilization and stored at −80°C before use.

### 2.2. Determination of Anthocyanins, Total Proteins, and Total Soluble Carbohydrate Contents in MAE

Separation of anthocyanins was conducted on Promosil C18 column (4.6 × 250 mm, 5 *μ*m) using an HPLC instrument (Thermo UltiMate 3000). Anthocyanin standards (J&K Chemicals, China) were used for quantitative determination. The contents of total proteins and total soluble carbohydrate in MAE were determined using the Bradford and the phenol-sulfuric acid method, respectively.

### 2.3. Cell Culture and Treatments

HepG2 cells were cultured in DMEM supplemented with 10% fetal bovine serum, 100 IU/mL penicillin, and 100 *μ*g/mL streptomycin at 37°C in a 5% CO_2_ atmosphere. After reaching 70–80% confluence, the cells were washed with PBS twice and incubated in the absence or presence of MAE with different concentrations for 24 h. The powder of MAE was dissolved in PBS buffer to produce the stock solution (10 mg/mL) and was freshly diluted in media without FBS to a certain concentration. And then, 300 *μ*M H_2_O_2_ was added into the media for another 4 h. Cells without H_2_O_2_ treatment were used as the negative control, and cells treated with glutathione (GSH, 1 mM) were used as the positive control.

### 2.4. MTT Assays

Cells were seeded into a 96-well plate, and MTT diluted with PBS at a final concentration of 0.5 mg/mL was added to each well after treatments. After 4 h of incubation at 37°C, the formazan precipitate was dissolved in 150 *μ*L DMSO and the absorbance was measured at 570 nm with a spectrophotometer [20]. In our studies, the treatment of HepG2 cells with MAE at 100 *μ*g/mL produced no detectable cell toxicity, which was consistent with previous research [21–23].

### 2.5. Glucose Consumption and Uptake Assays

The glucose consumption was estimated by the method of Lv et al. [24] with modification. Cells were seeded into a 96-well plate with five wells left as blanks. After H_2_O_2_ treatment, all media were removed and changed to the same. 24 h later, the glucose in the medium of each well was measured by a commercially available kit using the glucose oxidase method. 2 *μ*L medium was added into 200 *μ*L reaction agents, incubated for 20 min at 37°C, and then detected at 505 nm by a microplate reader (Molecular Devices, USA). Glucose consumption was calculated by the glucose concentrations in blank wells (no cell) minus glucose concentrations in plated wells. The MTT assay was used to adjust the glucose consumption. There were five replicates for each treatment and the experiment was repeated three times.

Cells with different treatments were exposed to 0.1 mM 2-NBDG and 100 nM insulin for 30 min at 37°C. Images were obtained using identical acquisition settings on a fluorescence microscope [25].

### 2.6. ROS and Superoxide Visualization

Cells were pretreated with MAE or GSH for 24 h, and then, 300 *μ*M H_2_O_2_ was added into the media for another 4 h. After that, all media were removed and changed to serum-free media loaded with H_2_DCFDA (for ROS measurement) or DHE (for superoxide determination) at 10 *μ*M for 30 min. Images were observed under a fluorescence microscope at identical exposure time. Densitometry analysis was performed using the Image-Pro Plus 6.0 software.

### 2.7. *C. elegans* Strains and Exposure

Strain N2 was provided by Dr. Du (Zhejiang University, China), and mutants *CF1038 [daf-16(mu86)I]*, *SPC167 [dvIs19 III*; *skn-1(lax120) IV]*, and *AY102 [pmk-1(km25) IV*; *acEx102]* were kindly provided by Dayong Wang (Southeast University, China). The strain was maintained at 20°C on a standard nematode growth medium (NGM) with *E. coli* OP50 as food resources.

Synchronized young adult worms were grown in NGM with FudR (5-fluoro-2′-deoxy-*β*-uridine, 50 *μ*g/mL) and OP50 containing different agents during the whole life span. A total of 30–40 young adults were cultured on each NGM and worms were counted every other day. Worms that did not move when gently touched with a platinum wire were considered dead. The whole experiments were repeated three times.

### 2.8. Analysis of Intracellular Lipofuscin and MDA

Worms were raised in NGM containing FudR and OP50 with or without paraquat and MAE. On the 10th day of adulthood, the intestinal autofluorescence by lipofuscin was analyzed. Twenty randomly selected worms from treatments were mounted onto microscope slides coated with 1% agarose and anesthetized with 5 mM levamisole. The autofluorescence of lipofuscin was captured with a fluorescence microscope (Leica, Wetzlar, Germany) using the DAPI filter set [26]. Densitometry analysis was performed using the Image-Pro Plus 6.0 software.

Worms with different treatments were washed from plates by M9 buffer and were ground in liquid nitrogen. Malondialdehyde (MDA) contents were determined by commercial kits (Nanjing Jiancheng, China).

### 2.9. Quantitation of PMK-1 and SKN-1 Expressions

Age-synchronized worms were treated with the MAE on the first day after hatching for 48 h and were then exposed to 1 mM paraquat for 24 h. After induction, expressions of PMK-1 and SKN-1 were measured directly through measuring the fluorescence intensity of the reporter protein GFP by fluorescence microscopy.

### 2.10. RNA Isolation and qPCR Analysis

Total RNA from worms was extracted using an RNAiso Plus kit (code D9108B, TaKaRa, Japan) according to the manufacturer's instructions. Real-time PCR was performed using the SYBR Green Kit (Roche) on the ABI Step One RT-PCR system. Primers designed with Primer Blast were listed in Table S1 available online at https://doi.org/10.1155/2017/7956158. GPD-1 was used for the reference gene.

### 2.11. Western Blot

Total protein extracts were prepared using the WB/IP lysis buffer (Beyotime Biotechnology, China). Cytoplasmic and nuclear protein extracts were prepared using a commercial kit (Sangon, China) according to the manufacturer's instructions. Equivalent amounts of protein from cell homogenates were subjected to SDS-PAGE and transferred to PVDF membranes. Membranes were probed with primary antibodies against p-JNK, JNK, p-p38, and p38 (Beyotime Biotechnology, China) and Nrf2, HO-1, FOXO1, and PGC-1*α* (Abcam). Membranes were detected with horseradish peroxidase-conjugated secondary antibodies using the ECL detection system. *β*-Actin (Beyotime Biotechnology, China) was used as the loading control. Densitometry analysis was performed using the Image-Pro Plus 6.0 software.

### 2.12. Statistical Analyses

Data are means ± SD. Statistical analyses of the data were performed with SPSS for Windows Version 11.5. One-way ANOVA analyses and Duncan's multiple-range test were used to detect statistical significances. The unpaired *t*-test was used to assess the significant differences between groups and differences were considered significant when *p* < 0.05.

## 3. Results

### 3.1. Determination and Analysis of Compositions in the Extract

**Figure 1:**
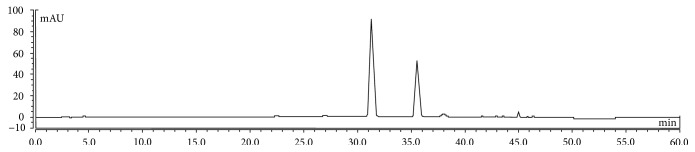
Identification and quantification of anthocyanins in MAE by HPLC analysis. The mobile phase A was 8.5% formic acid aqueous solution and the mobile phase B was formic acid/acetonitrile/methanol/water (8.5 : 22.5 : 22.5 : 41.5, *v*/*v*/*v*/*v*). A linear gradient program: 0–35 min from 7 to 25% B, 35–45 min from 25 to 65% B, 45-46 min from 65 to 100% B, and 46–50 min 100% B. The flow rate was 1.0 mL/min and the absorption spectrum was recorded at 535 nm. The column was operated at a temperature of 30°C. Three replicates were performed in the analysis.

HPLC analysis showed that the contents of anthocyanins in MAE were cyanidin-3-glucoside (47.2%), cyanidin-3-rutinoside (27.3%), and pelargonidin-3-glucoside (1.4%). Besides, there were 7.2% protein and 8.6% total carbohydrate in the extract (Figure 1 and Table 1).

### 3.2. MAE Abolished Both Glucose Consumption and Uptake Decreases in H_2_O_2_-Induced HepG2 Cells

**Figure 2:**
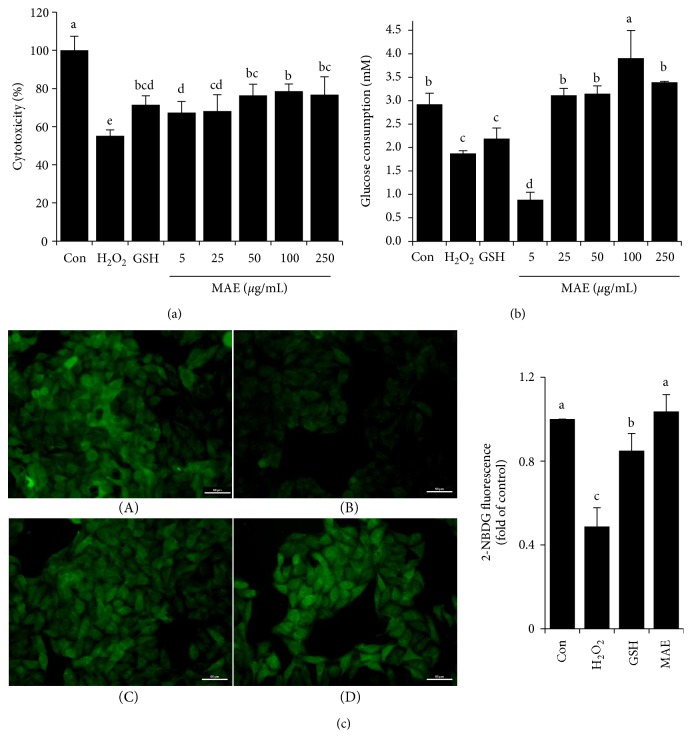
Effect of MAE on cytoprotection and glucose metabolism in H_2_O_2_-induced HepG2 cells. (a) Cell viability analyzed by MTT assays; (b) HepG2 cells were incubated with 5, 25, 50, 100, and 250 *μ*g/mL MAE or 1 mM GSH for 24 h and were exposed to H_2_O_2_ (300 *μ*M, 4 h). After that, all media were removed and changed to the same for glucose consumption measurement. Vertical lines represent standard deviations of five replicates. (c) Uptake of 2-NBDG into HepG2 cells. (A) control, (B) H_2_O_2_ (300 *μ*M, 4 h), (C) GSH (1 mM), and (D) MAE (100 *μ*g/mL). Densitometry analysis was performed using the Image-Pro Plus 6.0 software. Values with different letters above are significantly different, *p* < 0.05, one-way ANOVA test.

As shown in Figure 2(a), H_2_O_2_ incubation led to a significantly reduced cell viability, which was obviously recovered by GSH or MAE pretreatment, and the protective effect was dose-dependent around a range of concentrations. Compared to the control, an obvious decrease in glucose consumption and uptake was investigated in cells with 300 *μ*M H_2_O_2_ exposures for 4 h, indicating that insulin resistance happened in cells that suffered from oxidative damage. However, MAE significantly restored the glucose consumption by 65.6%, 67.6%, 107.8%, and 80.3% at 25, 50, 100, and 250 *μ*g/mL, respectively. GSH, as an antioxidant agent, also showed a recovery capacity on glucose consumption. Additionally, both GSH and MAE were able to avoid the inhibited glucose uptake caused by H_2_O_2_, showing similar results with glucose consumption (Figures 2(b) and 2(c)).

### 3.3. MAE Inhibited H_2_O_2_-Induced ROS and O_2_^−^ Generation in HepG2 Cells

**Figure 3:**
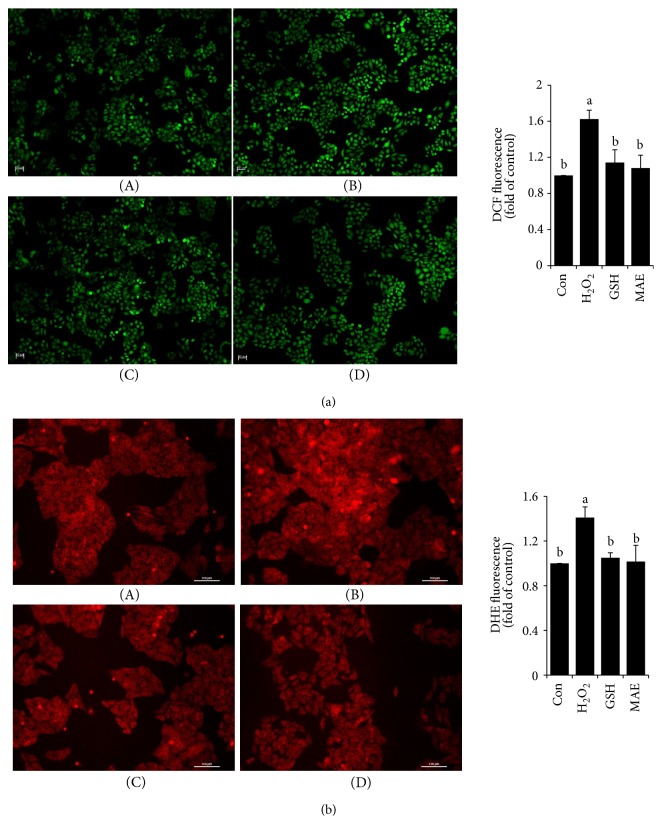
MAE reduced H_2_O_2_-induced intracellular ROS and O_2_^−^ levels in HepG2 cells. Cells with different treatments were incubated with H_2_DCFDA or DHE for (a) ROS and (b) O_2_^−^ analysis. The results are shown as representative microscopic scans: (A) control, (B) cells induced by H_2_O_2_ (300 *μ*M, 4 h), (C) GSH (1 mM), and (D) MAE (100 *μ*g/mL). The quantitative analysis of fluorescence from three independent replicates was used by the Image-Pro Plus 6.0 software. Values with different letters above are significantly different, *p* < 0.05, one-way ANOVA test.

An increase in antioxidant ability to inhibit ROS and O_2_^−^ generation is one of the strategies to prevent hepatotoxicity triggered by oxidative stress. Basal ROS and O_2_^−^ levels were increased by approximately 60% and 40%, respectively, in H_2_O_2_-treated HepG2 cells, while pretreatment with MAE (100 *μ*g/mL) significantly inhibited ROS and O_2_^−^ formations (Figure 3).

### 3.4. Effect of MAE on Protein Expressions of Nrf2 and MAPKs

**Figure 4:**
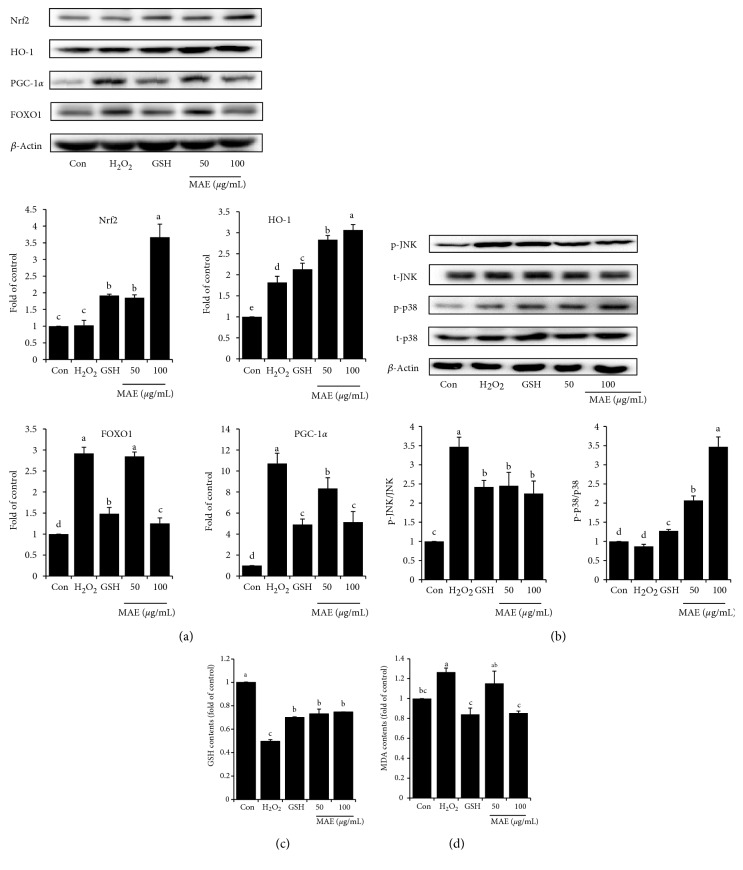
MAE treatment altered expressions of proteins associated with oxidative stress. (a) Protein levels of Nrf2, HO-1, PGC-1*α*, and FOXO1 in total cell extracts. Equal loading of Western blots was ensured by *β*-actin. (b) Protein levels of phosphorylated and total JNK and p38 in total cell extracts. Results were expressed as the ratio of phosphorylated/total protein levels. (c) GSH and (d) MDA contents determined by commercially available kits (NJJCBIO, China). Vertical lines represent standard deviations of three replicates. Values with different letters above are significantly different, *p* < 0.05, one-way ANOVA test.

Treatment with H_2_O_2_ for 4 h caused no change in Nrf2 expression but the HO-1 level was higher than that of the control. Nrf2 activation in HepG2 cells was induced by MAE, accompanied by the stimulation of HO-1. In parallel with this enhancement, GSH showed similar results with MAE, but less obvious change. Besides, H_2_O_2_ induced FOXO1 and PGC-1*α* expressions and MAE repressed these alterations (Figure 4(a)). The phosphorylation of JNK was increased while no significant difference in p-p38 between the control and H_2_O_2_-induced cells. Again, MAE prevented the phosphorylation of JNK and meanwhile triggered the phosphorylation of p38, which may result in Nrf2 activation (Figure 4(b)). What is more, MAE also recovered GSH content as well as suppressed MDA content of HepG2 cells challenged by H_2_O_2_ (Figures 4(c) and 4(d))_._

### 3.5. Effect of MAE on Nrf2, FOXO1, and PGC-1*α* Transcriptional Activities in HepG2 Cells

**Figure 5:**
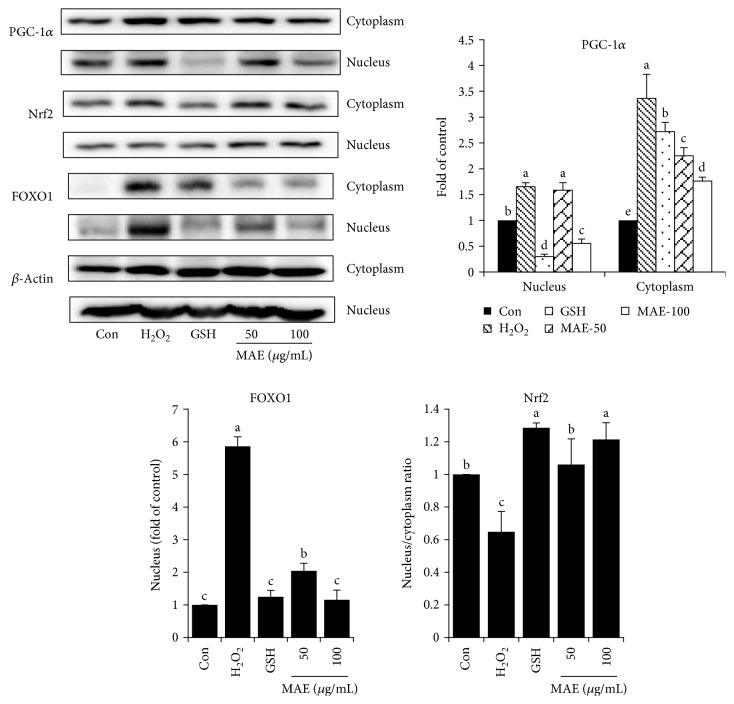
Effect of MAE on intracellular distribution of Nrf2, PGC-1*α*, and FOXO1 in HepG2 cells. The intensity of bands corresponding to Nrf2 in the nucleus was corrected by cytoplasmic protein levels to obtain relative measures of transcriptional activity among samples. FOXO1 expression level of the control in the cytoplasm is too low to be quantified. Vertical lines represent standard deviations of three replicates. Values with different letters above are significantly different, *p* < 0.05, one-way ANOVA test.

In order to measure activities of transcription factors, cytoplasmic and nuclear fractionations from cell extracts were used for Western blotting analysis. In comparison with the normal cells, levels of nuclear FOXO1 and PGC-1*α* were much higher in H_2_O_2_-induced treatments, suggesting that their activities were enhanced. Furthermore, the Nrf2 level in the ratio of the nucleus to the cytoplasm was obviously decreased by H_2_O_2._ In contrast, decreased levels of FOXO1 and PGC-1*α* as well as increased level of Nrf2 in the nucleus could be detected in cells pretreated with MAE, compared to those with the H_2_O_2_ treatment (Figure 5).

### 3.6. MAE Extended the Lifespan of *C. elegans*

**Figure 6:**
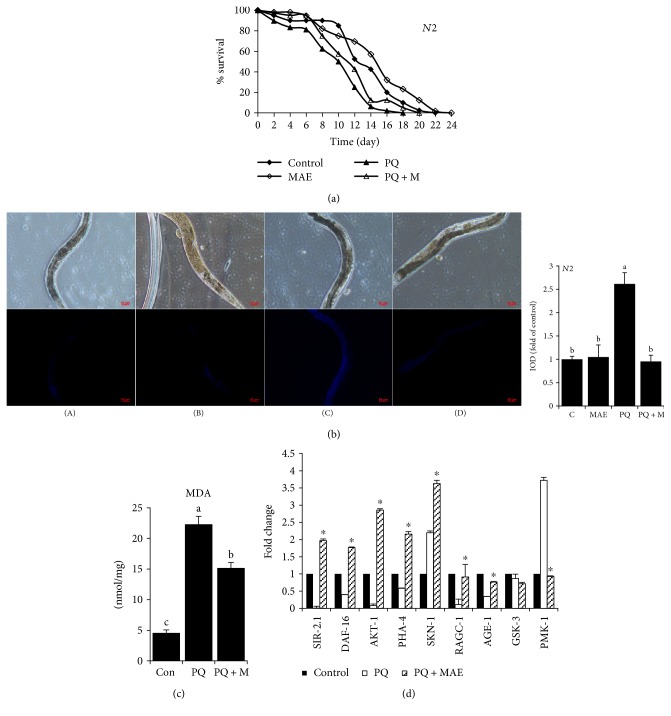
Protective effect of MAE on *C. elegans* under PQ exposure. (a) Lifespan of *N2* under different conditions. (b) On the 10th day of adulthood, the intestinal autofluorescence of lipofuscin was analyzed. (A) control, (B) MAE, (C) paraquat (1 mM), and (D) paraquat in the presence of MAE. Densitometry analysis was performed using the Image-Pro Plus 6.0 software. (c) MDA contents determined by a commercially available kit (NJJCBIO, China). Values with different letters above are significantly different, *p* < 0.05, one-way ANOVA test. (d) mRNA levels among control, paraquat (1 mM), and paraquat with MAE. ^∗^*p* < 0.05 versus paraquat.

Figure 6(a) demonstrated that exposure of worms to MAE increased lifespan compared to untreated ones. To test the protective ability against oxidative stress, we added paraquat into the medium, which could lead to a shortened life span. Our results also proved that the lifespan of *C. elegans* was shortened in the presence of paraquat, while MAE could partly reverse this damage, showing the longevity-promoting effect.

The nematode *Caenorhabditis elegans* contains autofluorescent lipofuscin granules, which can be quantitated by fluorescence spectroscopy. And this fluorescent material accumulated in animals with increasing age. Compared to controls, the paraquat-treated nematodes accumulated more lipofuscin at a greater rate. However, when paraquat-treated animals were exposed to 100 *μ*g/mL MAE, levels of lipofuscin were suppressed more than a half (Figure 6(b)). In addition, MAE alleviated the increased content of MDA in worms that was stimulated by paraquat (Figure 6(c)).

### 3.7. MAE Regulated Genes Involved in Longevity and Stress Response in *C. elegans*

**Figure 7:**
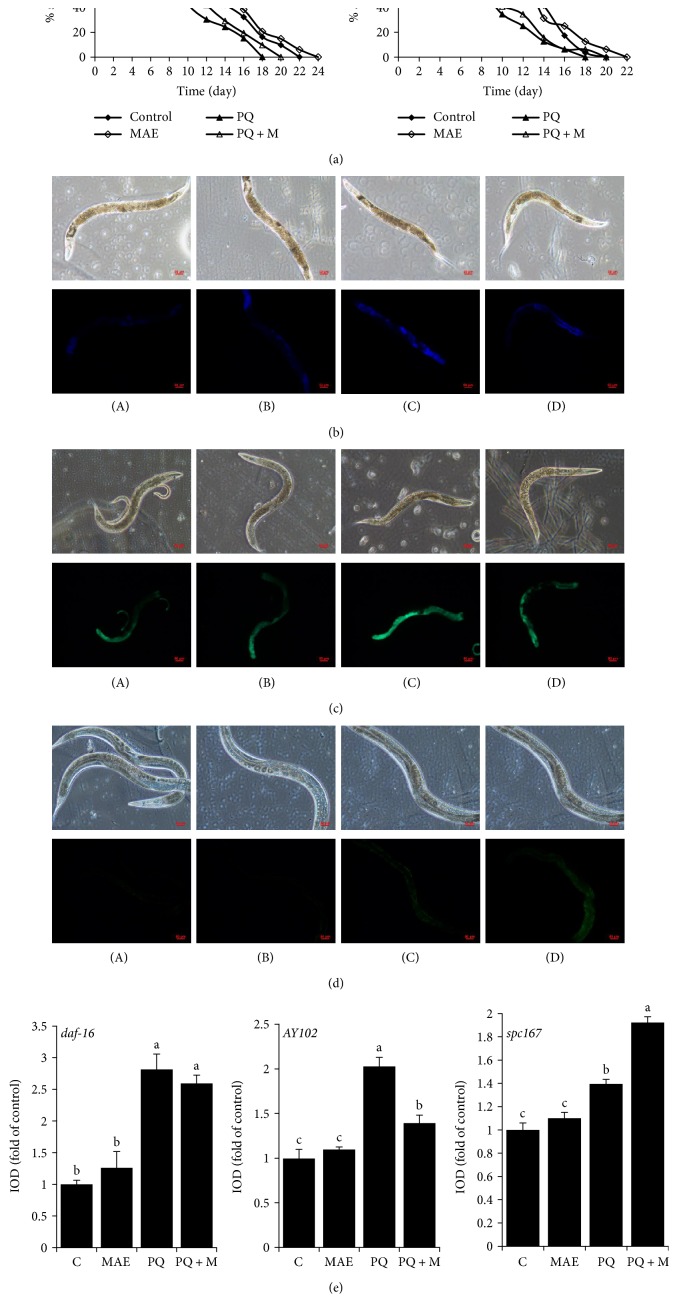
Effective assessment of MAE on oxidative protection in different mutants. (a) Lifespans of *daf-16* and *pmk-1*. (b) Lipofuscin levels of *daf-16* on the 10th day of adulthood. (c) PMK-1 expression in mutant *AY102* worms. (d) SKN-1 activity was imaged for GFP expression indicative of SKN-1 activation of the *gst-4p::gfp* reporter in mutant *SPC167* worms. (A) control, (B) MAE, (C) paraquat (1 mM), and (D) paraquat in the presence of MAE. (e) The quantitative analysis of fluorescence. Densitometry analysis was performed using the Image-Pro Plus 6.0 software. Values with different letters above are significantly different, *p* < 0.05, one-way ANOVA test.

Genes of SIR-2.1, DAF-16, AKT-1, PHA-4, RAGC-1, and AGE-1, which have a close relationship with aging or oxidative resistance, were all recovered with MAE treatment when worms suffered from paraquat. To confirm the molecular model of longevity in *C. elegans*, some mutants were used to evaluate the effects of MAE. In strains of *CF1038* (*daf-16* loss-of-function) and *AY102* (*pmk-1* partly loss-of-function), MAE failed to increase the survival rate under paraquat exposure (Figure 7(a)), in contrast to results obtained with N2 worms (Figure 6(a)). Furthermore, MAE also failed to suppress increased levels of lipofuscin in *CF1038* strain (Figure 7(b)). mRNA levels of PMK-1 and SKN-1 were activated after exposure to paraquat, and MAE suppressed PMK-1 activation but enhanced stimulation of SKN-1 (Figure 6(d)), which was consistent with the results of GFP expressions in *AY102* and *SPC167* mutants (Figures 7(c) and 7(d)). These findings indicated that MAE might perform its protective activity by modulation of the expression of stress-response genes, such as DAF-16, PMK-1, and SKN-1.

## 4. Discussion

Anthocyanins are key components in the human diet due to their frequent presence in plants, particularly dark-colored fruits, vegetables, and pigmented grains. During the past decade, anthocyanins have drawn increasing attention because they possess a powerful health-promoting property. In this study, anthocyanins extracted from mulberry fruit revealed three main ingredients recognized as cyanidin-3-glucoside, cyanidin-3-rutinoside, and pelargonidin-3-glucoside, which was consistent with previous research [11, 12] and accounted for more than 75% of the whole extract.

Apart from direct antioxidative reactivity, compounds such as certain polyphenols and flavonoids may also activate some intracellular signalling pathways like the Nrf2 pathway, to prolong the cellular defense response. In the present study, the lower Nrf2 protein is transferred into the nucleus in H_2_O_2_-treated cells, although levels have no significant difference in total cell extracts. MAE incubation not only leads to translocation of Nrf2 from the cytosol to the nucleus but also increases total cellular Nrf2 content, contributing to HO-1 and GSH level increases. Besides, distinct enhancement of p38 phosphorylation was investigated in cells treated with MAE. Considering p38 MAPK is vital in immune response and involved in Nrf2 activation in several cells [27], it seems probably that MAE can activate, at least in part, the p38 MAPK pathway, which in turn promotes Nrf2 activity. These results provide further evidence indicating that MAE enhances HO-1 expression by mediating Nrf2 translocation via p38 MAPK phosphorylation stimulation to scavenge H_2_O_2_-induced excessive free radicals.

Oxidative stress is the main contributor to diabetic complications, and H_2_O_2_ can result in ROS generation in the liver, which in turn causes hepatic insulin resistance [28, 29]. Studies have shown that cyanidin-3-glucoside isolated from mulberry fruit restrains high-glucose/H_2_O_2_-induced pancreatic *β*-cell death [30, 31]. In concurrence with previous studies, decreases in glucose consumption and uptake were observed in H_2_O_2_-induced HepG2 cells, meaning insulin resistance was formed. MAE treatments exhibit an evident increase in glucose consumption and glucose uptake in model cells, which means MAE can mitigate these adverse effects caused by H_2_O_2_. In the liver, glucose-6-phosphatase (G6Pase) and phosphoenolpyruvate carboxykinase (PEPCK) participate in committed steps of gluconeogenesis, playing vital roles in glucose homeostasis, and FOXO1 can regulate their expression levels by directly binding to their target DNA sequence [32]. Expression of PGC-1*α* and its target genes is implicated in hepatic gluconeogenesis, oxidative phosphorylation, and mitochondrial biogenesis [33]. What is more, there is also an interaction between PGC-1*α* and FOXO1 in the performance of insulin-regulated gluconeogenesis. Higher FOXO1 and PGC-1*α* expressions and transcriptional activities are investigated in H_2_O_2_-induced HepG2 cells while impaired by MAE. These changes may lead to PEPCK and G6Pase downregulation, causing a decrease in gluconeogenesis, and can result in antidiabetic.

JNK can be stimulated by ROS and cause insulin receptor substrates 1 (IRS1) serine phosphorylation to impair insulin signal transduction. Besides, activated JNK promotes FOXO1 activity by facilitating its movement to the nucleus [34]. A recent study has revealed that nuclear translocation of FOXO1 is rapidly induced by H_2_O_2_ but blocked by the JNK inhibitor [35]. On the contrary to p38, JNK upregulation is observed in H_2_O_2_-induced HepG2 cells while it is inhibited by MAE treatment, contributing to suppression of FOXO1 activity in response to the oxidative stress cells. Guo et al. reported that cyanidin-3-glucoside protected 3T3-L1 adipocytes against H_2_O_2_-induced insulin resistance by impairing JNK activation [36]. They also showed that beneficial effects of C3G in db/db mice were related to its inhibition on the JNK/FOXO1 pathway [37]. Given these findings along with our present data, we strongly suggest that these beneficial effects of MAE on insulin resistance improvement can be due to restrained upregulation of JNK, FOXO1, and PGC-1*α* in H_2_O_2_-induced HepG2 cells.

*C. elegans*, a highly advantageous organism, plays an important role in genetic and other in vivo research. To investigate whether the protective effect exhibited by MAE is evolutionarily conserved, we selected *C. elegans* for further experiments. Under the condition of oxidative stress, MAE as a powerful antioxidant has the capacity on cellular damage alleviation, explaining the extended lifespan of *C. elegans* treated with paraquat in the presence of MAE. Peixoto et al. [38] have demonstrated that stress resistance is increased and an aging-related marker is retarded in *C. elegans* by an anthocyanin-rich extract of acai, which is consistent with our results. AKT-1 and DAF-16/FOXO are vital factors in the insulin/IGF-1 pathway, associating with longevity in *C. elegans*. Chen et al. indicated that purple wheat that was rich in anthocyanin exhibited a lifespan-prolonging effect and was partly dependent on the activation of the transcription factor DAF-16/FOXO [39]. In this study, MAE also fails to extend the lifespan and lipofuscin accumulation in *daf-16* mutant, suggesting that DAF-16/FOXO is one of the targets of MAE to play an antioxidant activity. PMK-1/p38 has been found to phosphorylate SKN-1 directly to activate its activity, and the p38 MAPK pathway is highly related to oxidative stress response [40]. SKN-1, the *C. elegans* ortholog mammalian protein of Nrf2, participates in many regulatory pathways involved in different stresses, and it is also modulated by the insulin-like/IGF-1 pathway for the promotion of longevity. AY102 animals were developed by introducing the acEx102 extrachromosomal array into KU25 animals, resulting in intestinal PMK-1::GFP expression [41]. Because of *pmk-1* deletion, *AY102* animals were more susceptible to oxidative stress and MAE could not recover such damages, indicating that MAE may modulate PMK-1 activity in other tissues besides those of the intestine. GFP levels can be stimulated under some stresses in *SPC 167* worms [42], and our findings show that oxidative stress induces SKN-1 while MAE enhances such augment to prolong defensive reaction. However, it is necessary for more research to figure out the exact mechanism.

In conclusion, using in vitro studies, we suggest that MAE significantly mitigates H_2_O_2_-induced cytotoxicity by activating the MAPK-Nrf2 pathway in HepG2 cells. Additionally, MAE improves hepatic insulin resistance through inhibiting PGC-1*α* and FOXO1 transcriptional activity to decrease gluconeogenesis as well as suppressing JNK phosphorylation to enhance insulin signalling transduction. In vivo studies, we present evidence that MAE also extends the lifespan of *C. elegans* by activating DAF-16/FOXO, SKN-1/Nrf2, PMK-1/p38, and their downstream targets that are related to longevity and stress. These findings provide some new underlying mechanisms of mulberry anthocyanins on cytoprotection and antiaging, implying that MAE may serve as a strategy to help improve human health.

## Supplementary Material

Table S1. Sequence of primers used for qPCR analysis.

## Figures and Tables

**Table 1:** Quantification of anthocyanins, protein, and total soluble carbohydrate contents in MAE.

Fraction	Retention time (min)	Content (mg/g)
Cyanidin-3-glucoside	31.2	472 ± 9.23
Cyanidin-3-rutinoside	35.7	273 ± 5.12
Pelargonidin-3-glucoside	38.0	14 ± 0.29
Protein	—	72 ± 2.8
Total soluble carbohydrate	—	86 ± 3.6

Anthocyanin concentrations were determined by HPLC analysis. Protein content was determined by the Bradford method and measured at 595 nm. Total soluble carbohydrate was determined by the phenol-sulfuric acid method and measured at 490 nm. All values are mean ± SD. Three replicates were performed in the analysis.

## Abbreviations

MAE: Mulberry anthocyanin extract
IR: Insulin resistance
Con: Control
GSH: Glutathione
Nrf2: Nuclear factor erythroid 2-related factor 2
JNK: c-Jun N-terminal kinase
MAPKs: Mitogen-activated protein kinases
FOXO1: Forkhead box protein O1
PGC-1*α*: PPAR*γ* coactivator 1*α*
ROS: Reactive oxygen species
HO-1: Heme oxygenase-1
H_2_O_2_: Hydrogen peroxide
PQ: Paraquat
MDA: Malondialdehyde.
