# Supplementary material for: Mulberry Anthocyanin Extract Ameliorates Oxidative Damage in HepG2 Cells and Prolongs the Lifespan of Caenorhabditis elegans through MAPK and Nrf2 Pathways
Source: Oxid Med Cell Longev. 2017 Jun 21;2017:7956158. doi: 10.1155/2017/7956158 (PMC5497675; doi:10.1155/2017/7956158)
Supplement: Supplementary file 1 — Table S1. Sequence of primers used for qPCR analysis. [file 7956158.f1.docx]

| Gene name | Genbank accession | Primer sequence (5’-3’) |
| --- | --- | --- |
| GPD-1 | NM_063836 | CACTGGCGAAGGTTATCAA |
|  |  | GGAAACATCTGGTGTAGGGA |
| Sir-2.1 | NM_001268556 | AGGCAGAAACTCACAAACT |
|  |  | TGTAGAACGGAGCAGGATT |
| DAF-16 | NM_001313505 | AGCCAAGAAGAGGATAAAGG |
|  |  | AGAAACACGAGACGACGAT |
| AKT-1 | NM_001028476 | AGTCGGCAGAAGTTCGTCAG |
|  |  | CGTGTCCCGAAGATGGTTGA |
| PHA-4 | NM_001047651 | GAACCACGCAAGCACAGATG |
|  |  | CGGGTTGGTGGAGCTGTAAA |
| SKN-1 | NM_171347 | TTTTGCCTCCTCTCTTCTGGC |
|  |  | GGCGTATGGATGTTGGTGATG |
| RAGC-1 | NM_069786 | CGGACCCTGACGAGGATTTT |
|  |  | CCGCTTCTCTTGTGTCCCAT |
| PMK-1 | NM_068964 | TCCGACTCCACGAGAAGGAT |
|  |  | CACGATATGTACGACGGGCA |
| AGE-1 | NM_064061 | TCTGATTGCTGGACACGGAC |
|  |  | GCAGCGATCCTTTGCCATTC |
| GSK-3 | NM_060842 | TGCACGTCTTCCATCTGGTC |
|  |  | CACATCACCGCTTGTCGTTG |

Table S1. Sequence of primers used for qPCR analysis.
